# Developing ChatGPT’s Theory of Mind

**DOI:** 10.3389/frobt.2023.1189525

**Published:** 2023-05-30

**Authors:** Antonella Marchetti, Cinzia Di Dio, Angelo Cangelosi, Federico Manzi, Davide Massaro

**Affiliations:** ^1^ Research Unit on Theory of Mind, Department of Psychology, Università Cattolica del Sacro Cuore, Milan, Italy; ^2^ Research Unit on Psychology and Robotics in the Lifespan, Department of Psychology, Università Cattolica del Sacro Cuore, Milan, Italy; ^3^ Manchester Centre for Robotics and AI, University of Manchester, Manchester, United Kingdom

**Keywords:** ChatGPT, theory of mind, social perception, artificial intelligence, social cognition

The implementation of ChatGPT by OpenAI and the ensuing development of similar tools by other hi-tech companies have reignited the debate on the potential of artificial intelligence (AI) as a form of support in human activities. It seems increasingly likely that these “artificial agents” will soon become credible interlocutors for tasks in medium- to long-term interactions.

This technology has rekindled interest in psychology, reviving the metaphorical link between the human mind and AI. In developmental psychology, many start wondering if this conversational technology is capable of exhibiting a Theory of Mind (ToM), that is, the ability to interpret the behavior of others based on their mental states, such as emotions, goals, desires, and true and false beliefs.

ChatGPT has been proven capable of successfully passing language-based classical ToM tasks, including first-order meta-representations and socially ambiguous situations, such as those in the Strange Stories (Brunet-Gouet et al., 2023; Kosinski, 2023). How is this possible? Some clues come from Brunet-Gouet et al. (2023): “ChatGPT responses would not correspond to the natural responses of human subjects unless they were prompted to discuss all hypotheses and their probabilities” (p.9). This implicitly denounces ChatGPT’s tendency to violate the Gricean maxim of quantity by excessively leaving a flavor of artificiality in the response.

Verifying and confirming for ourselves what others have already observed, ChatGPT successfully passed the Sally–Anne test (1st-order; Wimmer and Perner, 1983), the Ice-Cream-Van task (2nd-order; Perner and Wimmer, 1985), the third-order false-belief task (Valle et al., 2015), and some Strange Stories, an advanced ToM task that deals with ambiguity in everyday life situations, where ambiguity requires reference to mental states in order to be resolved (i.e., a story of mixed emotions in which the protagonist is both sad about losing a race and happy for her friend who won it). Furthermore, we challenged ChatGPT by administering a faux pas story (Gregory et al., 2002), which could only be resolved if the underlying conversational implicature was understood. In the story, X unintentionally revealed to Y that X’s husband was organizing a surprise party for her, and ChatGPT succeeded in demonstrating its ability to capture linguistic cues even when the meaning was embedded. The answers to the test and justification questions were correct and argumentatively plausible. Then, we set off again to check the cross-linguistic validity of these results by administering the test in Italian. Interestingly, we found some evidence of hypermentalization (Bateman and Fonagy, 2015): “However, when Sally discovers that the marble is no longer there, she initially accuses Anne of taking it.” We readministered the classic Sally–Anne false belief two weeks apart to take note of any changes (Development? History of previous prompts?) in the responses. ChatGPT made a kind of unsolicited clarification by answering the test question as follows: “Typically, children between the ages of 3 and 5, like Sally and Anne in your story, have a limited knowledge of ToM, i.e., the understanding that other people may have beliefs and intentions that differ from their own.” This clarification adds nothing to the correct answer given previously. ChatGPT goes so far as to autonomously give the title “ball stolen by Anne” to the situation described, thus pursuing the hypermentalizing direction already taken. As a matter of fact, the possible reasons behind the ball’s displacement are multiple and not at all necessarily malevolent.

This raises a relevant question from two points of view. First, the variability found likely stems from the fact that the prevalent language for psychological literature is English and that during translation, some meanings give way to others, depending on the minority nature of the target language (Shatz et al., 2006). The second point of view concerns the cross-cultural validity of the use of ChatGPT. We believe that both points of view necessarily require an in-depth debate.

In addition to the various cross-linguistic nuances and consequent implications suggested previously, we further explored ChatGPT’s ToM mastering. What if we explicitly asked ChatGPT to put itself in someone else’s shoes, i.e., to use its knowledge about ToM to predict the responses to the first-order false-belief test as a 3-year-old? The request makes sense because ToM is, first and foremost, the ability to recognize that one’s own mental states may, in principle, be different from those of others and may not reflect reality. Interesting results emerged here, which outlined the interplay between ChatGPT’s adult-centric perspective and its dependence on the linguistic formulation of the questions. Specifically, we found that, when asked what it thinks an adult would respond to the question about first-order false belief and its justification, ChatGPT answered correctly. To the same question, asking what it thinks of a 3-year-old, ChatGPT incorrectly attributed a first-order ToM to the child based on an incorrect justification: “They (3-year-olds) may not understand the concept of object permanence and may assume that the object remains where it was last seen.” An error within an error (i.e., a wrong attribution of the false belief based on an incorrect justification). When asked the same question by replacing “how do you “think” with “pretend” to be a 3-year-old,” ChatGPT answered correctly, providing adequate justification. However, when asked to pretend to be a 4-year-old, it correctly answered the false-belief question, although providing a justification that undermined the validity of its correct answer by stating “children of this age may still have difficulty with the more complex aspects of Theory of Mind, such as understanding that beliefs can be false.” Understanding that beliefs can be false is, in fact, the prerequisite for the emergence of ToM. For humans, the variability of responses over development in the life-span as the wording of questions varies has been amply demonstrated (Siegal, 2013), as well as children’s mismatch between responses and justifications in false-belief tasks (Lombardi et al., 2018). However, it is surprising to find the same phenomenon in AI, which should be able to handle the same concepts conveyed by different linguistic forms. Paraphrasing Floridi’s (2023) statement “AI as agency without intelligence,” [sic.] we observe “ChatGPT’s ToM as ToM camouflage” (Corbett et al., 2021).

This fragility in the system becomes clear when comparing the development of natural ToM with the learning process of ChatGPT’s artificial ToM. Let us imagine a “child” (ChatGPT) being raised (trained) to understand the mind only through texts. How could we foster the development of its ToM? An approach would be by listening to the texts read by others and then having the child read texts by her/himself. Reading stories offers the opportunity to combine the knowledge of the language with the knowledge of the mind. This represents the first level of incorporation of cognition into a language, or vice-versa, and shows the inextricability between the two, providing the dyadic relational framework within which this inextricability can be contextualized through the reflective reading of the text. In addition, consider that such reading is often accompanied by visual experiences, i.e., images that link the reader to the states of the world that refer to the states of the mind described in the text. This complex relational experience constitutes the second level of incorporation or contextualization of meanings in the lived experience (the mediating role of the first-person knowledge with respect to the second-person knowledge that texts offer). However, in its text-only version of the world, ChatGPT cannot develop a sensorimotor grounded representation of its knowledge.

From an epistemological perspective, what kind of ToM developmental model is ChatGPT resembling? This question is not based on the assumption that the ToM of ChatGPT is analogous to the natural human ToM in architecture but only in surface behavior, following the same kind of analogical reasoning used to compare human cognition with computer information processing during the decades of dominance of the Human Information Processing (HIP) approach. After this necessary clarification, the usefulness of attempting to map explanations of ToM development in humans onto a technical system, such as ChatGPT, lies in the fact that humans are and will increasingly be engaged in verbal exchanges with conversational systems like ChatGPT. This necessarily implies that they will have to interface their own cognitive architecture devoted to ToM with the neural networks from which the ToM of artificial conversational agents is generated. A better understanding of the nature of this hybrid meeting between different (natural and artificial) ToMs will therefore help make the source from which artificial conversations originate more understandable, transparent, and reliable for humans. Returning to our question, i.e., which development model does ChatGPT’s ToM resemble? When asked, “Are you able to put yourself in other people’s shoes,” it replies “Yes, I am able to “simulate” other people’s mental states.” However, remember that it can fail to predict the response of another person endowed with lower cognitive capacities in the ToM test described previously. Therefore, it does not seem that the simulation theory (Harris, 1992) is the most appropriate model to describe ChatGPT’s ToM and neither is Bruner’s (Bruner, 1990) narrative thinking model, which envisages ToM developing in parallel with the construction of the self through cultural exchanges rooted in contexts. In addition, it does not appear that the associative deep learning model on which ChatGPT is based allows for developmental-stage jumps, as envisaged by the theory–theory model (Bartsch & Wellman, 1995). On the other hand, a purely verbal-linguistic modular model (Leslie et al., 2004) could well explain ChatGPT’s ToM development. The ChatGPT model remains deprived of connections within complex modular systems encompassing knowledge derived from other types of associative training inherent to language, e.g., prosody and rhythm, and even more to other forms of experience. The gap between the artificial and natural ToM in ChatGPT would be greatly reduced while remaining within the modular model, if the linguistic module were flanked by sensory modules of various types, allowing for the multidimensional access to information that pinpoints the natural development of ToM. This move toward multiple sources of knowledge could open up useful avenues for investment in research and future technological implementations. A further interesting direction of investigation that may usefully complement the one adopted in this contribution consists in investigating ToM and ChatGPT from a first-person perspective. Indeed, ToM skills are usually examined from a third-person perspective, in which ChatGPT is not an active participant in the events described but is rather a passive observer.

However, it is not surprising that ChatGPT makes mistakes; natural ToM makes mistakes as well. We would not be studying its development and various forms of impairment so passionately if mentalistic misunderstandings were not all but rare occurrences. This is not, in fact, the real interesting point of the discussion. On the contrary, if anything, error analysis can provide valuable insights to understand and more effectively implement a system that (without considering the huge ethical issues of its applications) already works admirably in certain respects. The point is to appropriately limit the analogies between the natural ToM and ChatGPT’s ToM, thus identifying explanatory models of the development of ToM as an emerging capacity (beyond its specific failures) for this form of AI. Luckily, as a serendipity effect, the human mind, either intentionally (think of novels) or unintentionally, inserts ToM crumbs/pieces/gems in texts written on ChatGPT. Through these findings and the questions we ask, we train ChatGPT, contributing to bringing out an ability—ToM—for which it has not been explicitly trained. Furthermore, the handling of exorbitant amounts of text has evolutionarily “called” ChatGPT’s particular form of associative learning to develop the best possible type of ToM, given the situation, to cope with human queries proposed and handled through text production. Here, perhaps the analogy with the natural ToM can be proposed, which developed in response to the need for social exchange, cooperation, and conflict management, in short, as an adaptive function.

In other words, ChatGPT’s ToM is of verbal intelligence, devoid of interchanges with the states of the world and related states of the mind, and thus forced to adhere to criteria of truth and validity of utterances in an exclusively self-referential manner. The opportunities to update its knowledge do come to it from the outside in the form of new texts and new questions from the users, but it lacks the capacity to suspend judgment in order to verify the comparative reliability of sources, the bona fides of questions, and, in short, to exercise critical thinking. Its ToM is the outcome of this process, and this is what it can return when called upon by its conversational partner. Moreover, as said, by updating its answers, ChatGPT ‘develops’ its ToM by sometimes making undue hypermentalizing inferences, performs from an adult-centric perspective, and changes its “mind” depending on how we phrase our questions. This is most likely due to a very rapid developmental acceleration with the possible undesirable side-effect of ChatGPT becoming unpredictable for a potential interlocutor who repeatedly questions it about the mind using different words for the same content (as humans typically do). This unpredictability happens in general with large pre-trained language models and some technical adjustments have been proposed (Mitchell et al., 2022). The possible effects of unpredictability in the management of mental states within interactions have been well-documented by both developmental and clinical research studies (Rolli, 2021). Let us think about what it might mean from the perspective of a conversational–educational–rehabilitative use of ChatGPT’s ToM with users in typical, atypical, or clinical conditions. For this reason, a promising scenario would be to implement individualized ways of managing interactions in order to guarantee—through conversational continuity and the fostering of an “episodic” rather than encyclopedic memory—stability and trustworthiness, which is fundamental for social exchanges. Finally, as for natural intelligence, given the promotion of civilized living, collective well-being and mental health, respect for minorities, and reduction of inequalities, ChatGPT must be trained in critical thinking and reflexivity. This cannot be fully achieved unless ChatGPT connects the language system and the world in the sense described previously because nothing human—and ToM less than ever—can be ascribed to the purely ungrounded language.
